# Utility of recombinant human TSH stimulation test in the follow-up of patients with differentiated thyroid cancer depending on basal thyroglobulin results

**DOI:** 10.1515/almed-2019-0017

**Published:** 2020-01-07

**Authors:** Amaia Sandúa, Monica Macias, Carolina Perdomo, Juan Carlos Galofre, Roser Ferrer, Estibaliz Alegre, Álvaro González

**Affiliations:** Service of Biochemistry, Clínica Universidad de Navarra, Pamplona, Spain; Department of Endocrinology, Clínica Universidad de Navarra, Pamplona, Spain; Instituto de Investigación Sanitaria de Navarra (IdiSNa), Pamplona, Spain; Hospital Universitario Vall d’Hebron, Barcelona, Spain

**Keywords:** differentiated thyroid cancer, rhTSH stimulation test, thyroglobulin, antithyroglobulin antibodies, thyroidectomy

## Abstract

**Background:**

Thyroglobulin (Tg) is fundamental for differentiated thyroid cancer (DTC) monitoring. Tg detection can be enhanced using recombinant human thyroid-stimulating hormone (TSH) (rhTSH). This study is aimed to evaluate the use of the rhTSH stimulation test when using a high-sensitivity Tg assay.

**Methods:**

We retrospectively studied 181 rhTSH tests from 114 patients with DTC and negative for antithyroglobulin antibodies (anti-TgAb). Image studies were performed in all cases. Serum Tg and anti-TgAb were measured using specific immunoassays.

**Results:**

rhTSH stimulation in patients with basal serum Tg (b-Tg) concentrations lower than 0.2 ng/mL always resulted in rhTSH-stimulated serum Tg (s-Tg) concentrations lower than 1.0 ng/mL and negative structural disease. In patients with b-Tg concentration between 0.2 and 1.0 ng/mL, s-Tg detected one patient (1/30) who showed biochemical incomplete response. Patients with negative images had lower s-Tg than those with nonspecific or abnormal findings (p<0.05). Receiver operating characteristic curve analysis of the s-Tg to detect altered images showed an area under the curve of 0.763 (p<0.05). With an s-Tg cutoff of 0.85 ng/mL, the sensitivity was 100%, decreasing to 96.15% with an s-Tg cutoff of 2 ng/mL.

**Conclusions:**

Patients with DTC with b-Tg concentrations equal or higher than 0.2 ng/mL can benefit from the rhTSH stimulation test.

## Introduction

Differentiated thyroid cancer (DTC) comprises less than 1% of malignant neoplasia, although it represents the most prevalent endocrine cancer, with an increasing incidence worldwide, being more frequent in middle-aged women [1]. Papillary histology corresponds to most cases (95%) and presents the best prognosis. Treatment decisions, based on a preoperative risk assessment [2], include surgery (lobectomy or total thyroidectomy), radioactive iodine ablation (RAI), and thyroid-stimulating hormone (TSH) suppressive therapy [2], [3]. Although DTC has a very good prognosis, there is a need for an active follow-up [1].

Thyroglobulin (Tg) is a protein synthetized only by thyrocytes and released by both normal and tumor cells [1], [3]. The complete removal of thyroid tissue results in decreasing serum Tg concentrations to an undetectable concentration. Consequently, Tg becomes the fundamental biomarker in DTC monitoring [2], [4] and a key factor in the assessment of the dynamic stratification of response to treatment [5]. In fact, the 2015 American Thyroid Association (ATA) guidelines classified patients’ response in accordance with basal serum Tg (b-Tg) and stimulated (s-Tg) serum Tg concentrations [2]. However, quantifying serum Tg is nowadays technically challenging mainly because of variations in the functional sensitivity of the different assays [6], [7] and the potential presence of interferences, especially from anti-Tg antibodies (anti-TgAb) [8], [9], [10].

The concentration of circulating Tg parallels tumor burden. Therefore, it is crucial to enhance the sensitivity of Tg measurement to detect persistent or recurrent disease as soon as possible. Because Tg production is stimulated by TSH, serum Tg concentrations can increase either by inducing hypothyroidism by withdrawing thyroid hormone replacement or by administering recombinant human TSH (rhTSH) [11]. Stimulation with rhTSH (Thyrogen®) is an alternative to thyroxine withdrawal that avoids hypothyroidism morbidity [12].

rhTSH stimulation is an expensive test that lasts up to five days, with blood drawing in three different days. The test was of great use when using old Tg measurement methods with low sensitivity, which ranged between 0.5 and 1.0 ng/mL [10], [13]. However, more sensitive Tg measurement methods have improved the functional sensitivity to less than 0.2 ng/mL [9], making the use of rhTSH Tg stimulation test controversial for patients classification [2], [14]. rhTSH stimulation is currently used in numerous settings, and even recently, a wide consensus from the European group claimed the use of the rhTSH test in patients with medium or high risk or recurrence [15]. While most consensus was achieved to avoid stimulation test when b-Tg was in the limit of detection [6], [14], few data are available in those patients with values in the categories of indeterminate or biochemical incomplete responses. This study is aimed to assess the use of the rhTSH stimulation test when using a high-sensitivity Tg assay. A second aim of this study is to evaluate if the laboratory can help selecting patients in whom the rhTSH stimulus can retrieve relevant information.

## Materials and methods

### Patients

We retrospectively reviewed 181 rhTSH stimulation tests performed during the follow-up of 114 patients with DTC (mean age = 45.7 ± 14.3 years; 73% female). Up to 102 patients had papillary cancer, and 12 had follicular cancer. Inclusion criteria were patients with DTC previously treated by total thyroidectomy and RAI therapy. The exclusion criterion was the detection of anti-TgAb at baseline, to avoid erroneous serum Tg measurements. Thyroid cancer staging was performed in accordance with the American Joint Committee on Cancer and Union Internationale Contre le Cancer (AJCC-UICC) guidelines 8th edition [16]. The local Ethics Committee approved the study.

### Immunoassay

Serum TSH was measured using an electrochemiluminescence immunoassay in a Cobas 8000 (Roche Diagnostics, Mannheim, Germany). The manufacturer’s reference concentrations ranged from 0.27 to 4.20 mIU/L, with a claimed functional sensitivity of 0.005 mIU/L.

Tg was measured using a chemiluminescence immunoassay in an Access II (Beckman Coulter, Nyon, Switzerland). This method is standardized against CRM 457 reference material. The claimed detection limit and functional sensitivity were 0.01 ng/mL and 0.1 ng/mL, respectively [17].

Anti-TgAb were measured using a chemiluminescence immunoassay in an Access II (Beckman Coulter). Positivity was defined when anti-TgAb was detectable (limit of detection: 0.9 IU/mL).

All immunoassays were performed in the same laboratory, and methods were not changed throughout the study.

### rhTSH stimulation

The rhTSH stimulation test was performed using rhTSH injection (Thyrogen®, Genzyme, Cambridge, MA, USA) [18]. Briefly, after obtaining a blood sample for b-Tg, anti-TgAb, and TSH quantification, Thyrogen® (0.9 mg) was injected intramuscularly on two consecutive days. To check if the stimulation was correct, serum TSH concentration was measured on day 3. On the fifth day after the stimulus, s-Tg and anti-TgAb were measured again.

### Definition of response to treatment

The level of response to initial therapy was considered according to ATA thyroid cancer guidelines (2015) [2]: (a) excellent if b-Tg concentration was lower than 0.2 ng/mL or s-Tg <1.0 ng/mL; (b) indeterminate if b-Tg concentration was ≥0.2 and <1.0 ng/mL or s-Tg between ≥1 and <10 ng/ml; and (c) biochemical incomplete if b-Tg concentration was ≥1.0 ng/mL or s-Tg ≥10 ng/mL.

Patients were classified as per b-Tg into group A if Tg <0.2 ng/mL (excellent response), group B if 0.2 ≤Tg < 1.0 ng/mL (indeterminate response), and group C if Tg ≥1.0 ng/mL (incomplete response).

### Image studies

All the rhTSH stimulation tests were accompanied by image studies selected as per the endocrinologist criterion. All patients underwent ultrasonography that was combined in some patients with computed tomography scan, magnetic resonance imaging, or functional nuclear medicine imaging for searching potential local or distant metastases.

### Statistical analysis

Statistical analysis was performed using GraphPad Prism, version 6.07, (La Jolla, CA, USA). Non-Gaussian distribution of data was assessed using the Kolmogorov–Smirnov normality test. Data were expressed as median and interquartile range. Comparison between groups was performed using the Kruskal–Wallis test followed by the Dunn’s multiple comparisons test. Correlation was studied using the Spearman’s test. A Chi square test was used to analyze relationship between groups. Receiver operating characteristic (ROC) curves were constructed to determine the cutoff for better sensitivity. Concordance was evaluated with the Cohen's kappa index, which can be interpreted as follows: 0.81–1.00, very good; 0.61–0.80, good; 0.41–0.60, moderate; 0.21–0.40, slight [19]. A two-tailed p‐value of <0.05 was considered to be statistically significant.

## Results

### Basal and rhTSH-stimulated thyroglobulin

Following ATA guidelines [2], cases were divided as per b-Tg into three groups (Table 1): group A (76 cases, 73 patients); group B (70 cases, 30 patients), and group C (35 cases, 20 patients). Median serum TSH concentration measured at day 3 of stimulation was 142 mIU/L (Q1–Q3: 115–185 mIU/L). As expected, there was no relationship between TSH and either b-Tg or s-Tg.

**Table 1:** Concentration of thyroglobulin at baseline and after 5 days of stimulation with rhTSH.

Group	Basal Tg (biochemical response in accordance with ATA 2015 guidelines [2])	Patients, n	Cases, n	Median basal Tg (interquartile range)	Median post-rhTSH Tg (interquartile range)	Median fold concentration change (interquartile range)
A	<0.2 ng/mL (excellent)	73	76	0.1 ng/mL (functional sensitivity)	0.1 ng/mL (0.1–0.2 ng/mL)	
B	≥0.2 and <1.0 ng/mL (indeterminate)	30	70	0.40 ng/mL (0.37–0.60 ng/mL)	3.9 ng/mL (1.8–5.3 ng/mL)	6.9 (2.9–11.4)
C	≥1.0 ng/mL (incomplete)	20	35	2.6 ng/mL (1.48–3.63 ng/mL)	12.4 ng/mL (6.6–25.8 ng/mL)	4.29 (2.73–5.85)

ATA, American Thyroid Association; rhTSH, recombinant human thyroid-stimulating hormone; Tg, thyroglobulin.

**Figure 1:**
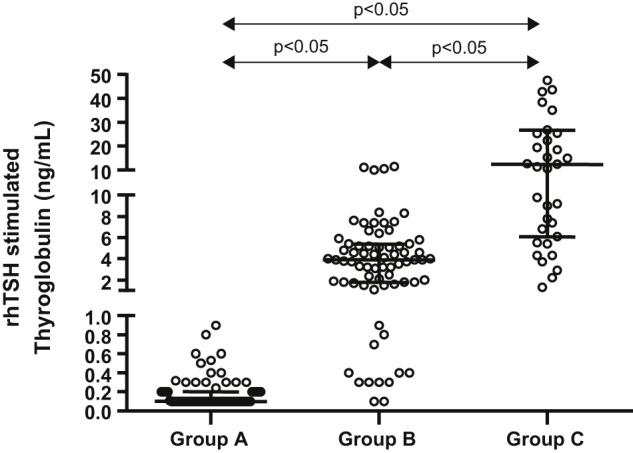
rhTSH-stimulated thyroglobulin levels according to basal thyroglobulin (Tg) concentrations. Patients were classified as per basal Tg into group A if Tg <0.2 ng/mL, group B if 0.2 ≤ Tg < 1.0 ng/mL, and group C if Tg ≥1.0 ng/mL. rhTSH, recombinant human thyroid-stimulating hormone.

Stimulation with rhTSH in group A patients always resulted in s-Tg concentrations lower than 1.0 ng/mL (Tables 1 and 2), which also corresponds to excellent response in accordance with the dynamic stratification assessment of the ATA. From 76 tests performed, there was an absence of Tg increase in 48 of them, while in the other 28, s-Tg increased, but only six of them showed concentrations between 0.5 and 0.9 ng/mL (Figure 1).

**Table 2:** Thyroglobulin concentrations and image studies regarding patient response to treatment (ATA 2015 guidelines [2]).

	rhTSH-stimulated thyroglobulin			Image		
	Excellent response	Indeterminate response	Biochemical incomplete response	Negative	Indeterminate	Structural disease
Basal thyroglobulin						
Excellent response	76	0	0	76	0	0
Indeterminate response	12	54	4	44	17	9
Biochemical Incomplete response	0	15	20	9	13	13
Image						
Negative	87	35	7			
Indeterminate	1	23	6			
Structural disease	0	11	11			

ATA, American Thyroid Association.

Among patients with indeterminate response (group B), we observed increases lower than 100% in 7 different patients, and b-Tg was lower than 0.4 ng/mL in all of them. There was a correlation between b-Tg and s-Tg (*r* = 0.394; p<0.01). s-Tg was statistically higher than that of the previous group (p<0.05; Figure 1). In 54 cases (76%), s-Tg resulted in biochemical indeterminate response (s-Tg ≥ 1 and <10 ng/mL), but in other 12 cases (18.3%), s-Tg resulted in excellent response (s-Tg <1.0 ng/mL). Only four cases, all of them from the same patient resulted in an s-Tg higher than 10 ng/mL.

Finally, regarding patients with incomplete response (group C), no statistically significant correlation was observed between b-Tg and s-Tg concentrations. s-Tg was significantly higher in this group than in the other two groups, with regard to b-Tg (p<0.05; Figure 1). In 15 cases, s-Tg resulted in indeterminate response (s-Tg <10 ng/mL), while in other 20 cases, s-Tg resulted in biochemical incomplete response. In three cases, corresponding to three different patients, b-Tg concentration was higher than 10 ng/mL. As expected, their corresponding s-Tg concentrations were higher than 10 ng/mL too.

There was a concordance for biochemical response classification between b-Tg and s-Tg results (kappa index = 0.732; 95% confidence interval [CI] = 0.645–0.818) when considering all the patients. However, when excluding those cases in which b-Tg was below 0.2 ng/mL (group A), the concordance decreased to moderate (kappa index = 0.392; 95% CI = 0.213–0.572).

### Basal and rhTSH-stimulated thyroglobulin in relation to image studies

Image studies were negative in all cases when b-Tg was lower than 0.2 ng/mL (group A, Table 2). Within group B, images were undetermined in 24% (17) of the cases and indicated persistent disease in other 13% (9), whereas 63% (44) had negative image studies. Finally, in group C, image studies were negative in 26% (9), indeterminate in 37% (13), and with structural incomplete response in the remainder 37% (13) of the cases. In those cases in which b-Tg was higher than 10 ng/mL, image studies showed structural incomplete response. Concordance between image studies and b-Tg was low (kappa index = 0.324; 95% CI = 0.207–0.441).

In group B, if s-Tg indicated excellent response (12 cases), all imaging studies were negative. If s-Tg indicated indeterminate response (54 cases), imaging studies were negative in 59% of them and indeterminate in the 28% of them and showed persistent disease in 13%. In addition, if s-Tg indicated biochemical incomplete response (4 cases), images were negative or indeterminate in 50% of the cases and showed persistent disease in the other 50%.

In group C, if s-Tg indicated indeterminate response (15 cases), image studies were negative or indeterminate in the 73% of them and showed persistent disease in 27%, whereas if s-Tg indicated biochemical incomplete response (20 cases), images were negative or indeterminate in 55% of the cases and showed persistent disease in the other 45%.

**Figure 2:**
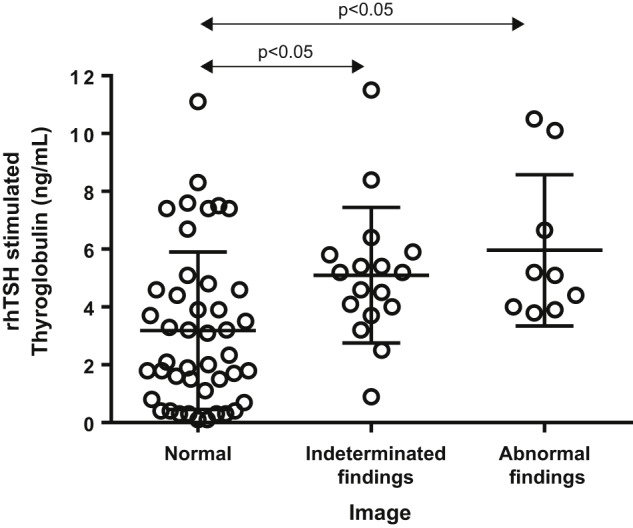
rhTSH-stimulated thyroglobulin levels as per image findings in the group of cases with basal thyroglobulin ≥0.2 ng/mL and <1.0 ng/mL (group B). rhTSH, recombinant human thyroid-stimulating hormone.

**Figure 3:**
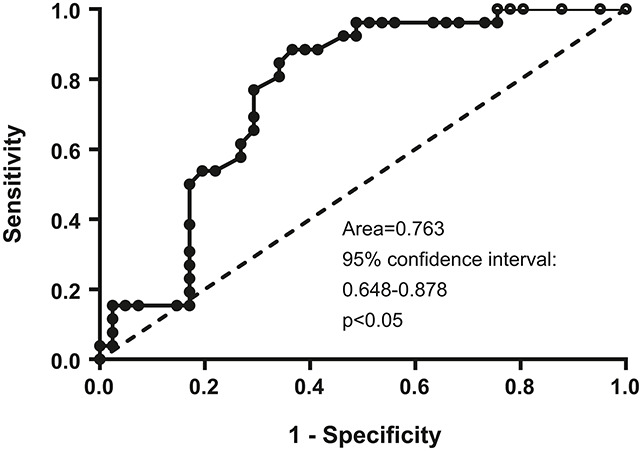
ROC curve analysis of rhTSH-stimulated thyroglobulin for abnormal image findings in cases with basal thyroglobulin ≥0.2 ng/mL and <1.0 ng/mL. rhTSH, recombinant human thyroid-stimulating hormone; ROC, receiver operating characteristic.

Within group B patients, no difference in b-Tg concentration was observed in accordance with image studies (data not shown). However, those with negative images had significantly lower s-Tg (median s-Tg = 2.2 ng/mL; Q1–Q3 = 0.9–4.6 ng/mL) than those with indeterminate (median s-Tg = 5.2 ng/mL; Q1–Q3 = 3.2–5.9 ng/mL; p<0.05) or abnormal findings (median s-Tg = 5.1 ng/mL; Q1–Q3 = 3.9–8.4 ng/mL; p<0.05) (Figure 2). Only in one patient with structurally persistent disease in the imaging studies, s-Tg concentrations were higher than 10 ng/mL. In this group B, ROC analysis of the s-Tg to identify altered images resulted in an area under the curve of 0.763 (95% CI: 0.6482–0.8780; p<0.05) (Figure 3). Using an s-Tg cutoff of 0.85 ng/mL the sensitivity to detect altered images was 100% (no false negatives), although with a specificity of 24%, while using a cutoff of 2 ng/mL, the sensitivity was 96.15%, although with a specificity of 46%.

## Discussion

In patients with DTC treated by total thyroidectomy in addition to RAI, the rhTSH stimulation test has been used to identify persistent or recurrent disease or to reclassify the risk of recurrence [3]. Our results show that when using highly sensitive assays, if b-Tg concentrations are within the limit of sensitivity, then s-Tg will always be below 1.0 ng/mL, which represents an excellent response too [2], [14], [20]; thus, it can exclude the necessity of a Tg stimulation test [21], [22]. Pacini et al. [23] indicated that b-Tg in the limit of detection or minimal detectable s-Tg with negative anti-TgAb can reliably identify disease-free patients. Furthermore, in our hands, all these patients had negative images and, consequently, an excellent response to treatment. In line with these results, Verburg et al. [24] recently reported that low or undetectable b-Tg concentrations make neck ultrasound unnecessary. If b-Tg ≥1.0 ng/mL, rhTSH stimulation does not seem to add additional information relating to ATA classification of biochemical risk assessment [2]. However, in these cases, it would be interesting to know if s-Tg concentration is able to differentiate between local or distant metastasis. Further studies would be necessary to investigate this point.

Group B is considered a “grey zone” in which rhTSH-stimulated Tg testing can provide thoughtful information [6]. Some authors have found recurrent disease in 12.5% of patients with low-risk DTC and with b-Tg concentrations higher than 0.15 ng/mL [25]. In some patients with b-Tg between 0.2 and 1.0 ng/mL, concentrations of s-Tg can result in a reclassification of patients as biochemical incomplete response, implying the need for consequential clinical decisions, such as a closer follow-up by careful imaging studies or new therapeutic procedures. All patients with s-Tg concentration lower than 0.85 ng/mL had negative images and thus no evidence of structural disease. This result is similar to those obtained by others, in which s-Tg concentrations lower than 0.5 ng/mL resulted in 98% likelihood of identifying patients completely free of tumor [13].

We have found a correlation between b-Tg and s-Tg only in group B. This is in contrast with previous reports that found a correlation between b-Tg and s-Tg across a wide range of b-Tg (0.05–1,000 ng/mL) [11]. The differences could be due to methodology, the type of patients, and the fact that these authors quantified s-Tg after 72 h.

All conclusions are drawn on the premise that endogenous anti-TgAb are negative. It is well known that anti-TgAb can interfere negatively in immunoassays decreasing Tg concentration measured [6], [26]. However, negative anti-TgAb do not exclude this potential interference as anti-TgAb can be missed because their detection is assay dependent [8], [9]. We should also consider the possible existence of a poorly differentiated aggressive tumor with limited Tg synthesis, resulting in low/negative b-Tg [6].

**Figure 4:**
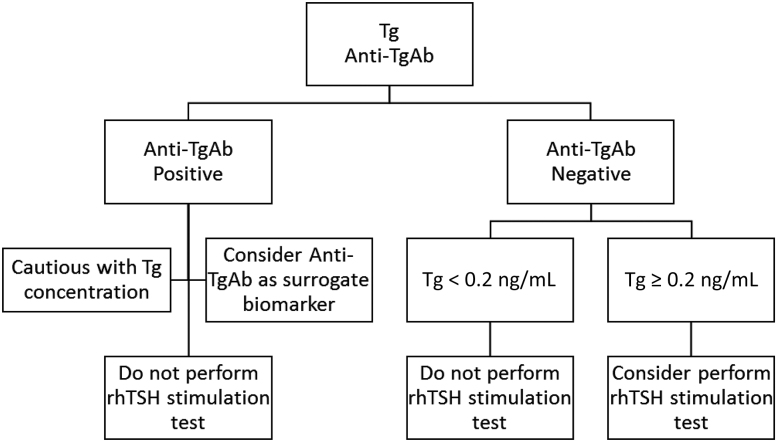
Proposed flowchart to select from the laboratory those patients who could eventually benefit from rhTSH stimulation test. rhTSH, recombinant human thyroid-stimulating hormone.

From a laboratory point of view, we suggest the following protocol in case an rhTSH-stimulated Tg is proposed (Figure 4): First, perform b-Tg test following a method with a functional sensitivity of 0.2 ng/mL or lower [6], as well as an anti-TgAb test. In case of anti-TgAb positivity, consider not performing the stimulation test because Tg quantification cannot be accurate, producing unreliable data because of this interference [4]. In these cases, anti-TgAb can be considered as a surrogate biomarker [3], [9]. In case of negative antibodies and b-Tg <0.2 ng/mL, do not perform rhTSH stimulation test, as this would not add any clinical valuable information. If b-Tg is ≥ 0.2 ng/mL, then rhTSH stimulation test could be performed. A similar flowchart was proposed by Giovanella et al. [9], although without considering anti-TgAb measurement, that we consider essential to avoid potential interferences. With this simple protocol, the number of rhTSH stimulation tests could be reduced significantly to only those cases in which it could be potentially beneficial, saving resources, and avoiding distress to patients.

In summary, we show here that some patients with DTC with b-Tg concentration lower than 0.2 ng/mL do not benefit from rhTSH stimulation test, while the test could be of interest in the other groups. To select this group of patients, the laboratory role is important as it produces the initial results. Communication between the laboratory and clinicians is therefore essential to develop the algorithm proposed.
